# Free-standing millimetre-long Bi_2_Te_3_ sub-micron belts catalyzed by TiO_2_ nanoparticles

**DOI:** 10.1186/s11671-016-1510-x

**Published:** 2016-06-24

**Authors:** Piet Schönherr, Fengyu Zhang, Danny Kojda, Rüdiger Mitdank, Martin Albrecht, Saskia F. Fischer, Thorsten Hesjedal

**Affiliations:** Clarendon Laboratory, Department of Physics, University of Oxford, Parks Road, Oxford, OX1 3PU UK; Humboldt-Universität zu Berlin, Newtonstr. 15, Berlin, 12489 Germany; University of Science and Technology of China, Jinzhai Rd. 96, Hefei, 230026 China; Leibniz-Institut für Kristallzüchtung - IKZ, Berlin, 12489 Germany

**Keywords:** Nanowires, Topological insulators, Temperature-dependent conductivity, Bismuth telluride

## Abstract

Physical vapour deposition (PVD) is used to grow millimetre-long Bi_2_Te_3_ sub-micron belts catalysed by TiO_2_ nanoparticles. The catalytic efficiency of TiO_2_ nanoparticles for the nanostructure growth is compared with the catalyst-free growth employing scanning electron microscopy. The catalyst-coated and catalyst-free substrates are arranged side-by-side, and overgrown at the same time, to assure identical growth conditions in the PVD furnace. It is found that the catalyst enhances the yield of the belts. Very long belts were achieved with a growth rate of 28 nm/min. A ∼1-mm-long belt with a rectangular cross section was obtained after 8 h of growth. The thickness and width were determined by atomic force microscopy, and their ratio is ∼1:10. The chemical composition was determined to be stoichiometric Bi_2_Te_3_ using energy-dispersive X-ray spectroscopy. Temperature-dependent conductivity measurements show a characteristic increase of the conductivity at low temperatures. The room temperature conductivity of 0.20 × 10^5^ S m ^−1^ indicates an excellent sample quality.

## Background

Bi_2_Te_3_ is a well-known thermoelectric and a topological insulator (TI) [1]. Interest in thermoelectrics is fuelled by the potential to generate power from waste heat [2, 3]. The thermoelectric efficiency is quantified by the figure of merit *ZT* which is a function of the electrical and thermal conductivity and the Seebeck coefficient of the thermoelectric material. Single-crystalline quasi-one dimensional structures on the nano- and sub-micron level are particularly suited to study surface effects such as morphological features or TI-based surface transport which is enhanced relatively due to the high surface-to-volume ratio [4, 5]. The topologically protected surface transport emerges as a result of strong spin-orbit coupling in Bi_2_Te_3_ and other materials [6]. The surface state is formed by a single Dirac cone with linear dispersion and has attracted great interest in the last decade [7]. It provides spin-momentum-locked electronic transport on the surface whilst the bulk of the material is a trivial insulator. In Bi_2_Te_3_, the bulk contribution to the total charge transport is very high which makes it challenging to characterize the topological surface state (TSS). It is one of the challenges in the field to overcome this hurdle by producing intrinsic materials with a high surface-to-volume ratio, such as single-crystalline nanowires, to effectively suppress the relative bulk contribution [8, 9].

The unit cell of Bi_2_Te_3_ consists of three quintuple layers (QLs) with the stacking sequence Te-Bi-Te-Bi-Te. Bi_2_Te_3_ nanowires grow parallel to these layers [10]. Synthesis techniques include solvothermal growth [10], molecular beam epitaxy [11], on-film formation [12], and physical vapour deposition (PVD) [13], among others [14]. However, the synthesized structures are often heterogeneous and short, as there are, e.g. platelets growing alongside wires which are less than some 10 *μ*m in length. There is a profound interest in long Bi_2_Te_3_ nanowires for three reasons: (i) They enable the observation of pronounced Shubnikov-de Haas oscillations as seen in long Bi_2_Se_3_ nanowires [5]; (ii) they offer the possibility to combine multiple devices on a single nanowire [15]; and (iii) long nanowires are an interesting building block for sensors that require few high-aspect electrodes over a wide area without the need of high spatial resolution [16].

Previous work on vapour-liquid-solid-grown (VLS-grown) Bi_2_Te_3_ nanowires was limited to quasi-four-point-probe measurements due to the short length of the nanowires [17]. The influence of contact resistance could not be fully excluded. Andzane et al. demonstrated four-point-probe measurements on Bi_2_Te_3_ nanobelts that were synthesized in a two-step process [18]. Here, we report the one-step synthesis of free-standing millimetre-long Bi_2_Te_3_ sub-micron belts by PVD. The growth yield is increased by an unusual catalyst for nanostructure growth, namely TiO_2_, which was reported to outperform Au catalysts in the growth of Sb-doped Bi_2_Se_3_ nanowires [19]. Four separated contacts are prepared by standard laser lithography to extract the temperature-dependent conductivity.

## Methods

The PVD growth was carried out in a Nabertherm B180 horizontal tube furnace (Lilienthal, Germany) under constant nitrogen flow of 300 sccm at atmospheric pressure using Bi_2_Te_3_ powder as a precursor. The furnace was flushed with nitrogen several times after loading Si(100) substrates (downstream) and the Bi_2_Te_3_ precursor (upstream) into quartz boats. Then, the oven was ramped to the growth temperature of 600 °C and held constant for a growth time of typically 1 h. The samples were removed after the furnace cooled down to room temperature and subsequently analysed by scanning electron microscopy (SEM), energy-dispersive X-ray spectroscopy (EDX), and atomic force microscopy (AFM). Individual belts were placed on a silicon wafer with 300 nm of SiO_2_ field oxide by a mechanical transfer method that provides sub-micrometer precision [20]. Four-point contacts were made by standard laser photolithography (using AZ3007 photoresist). After writing the pattern, the sample was H_2_-plasma cleaned at 100 W for 90 s. A 50 nm layer of Au was sputter-deposited and subsequently lifted off. The devices were glued into chip carriers using silver paint and wire bonded using Al wires. Temperature-dependent resistance curves *R*(*T*) were measured by current-voltage sweeps in four-point configuration (using a Keithley 6221 current source and 2182A nanovoltmeter). The samples were kept in a He atmosphere at ambient pressure. Subsequent to the transport measurements, cross sections were obtained using a Nova 600 NanoLab (FEI). First, the belt is covered with a platinum layer deposited at an electron voltage of 5 kV and a beam current of 0.4 nA. The platinum layer serves as protection layer and as thermal bridge to sink the heat during the focused ion beam etching. A gallium ion current of 50 pA is used at a voltage of 10 kV for etching.

## Results and discussion

**Fig. 1:**
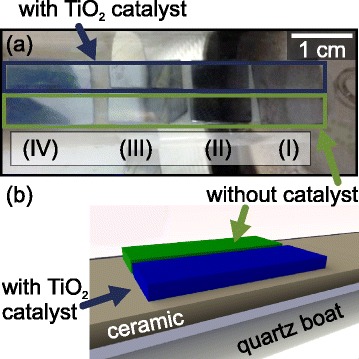
Substrate arrangement. **a** Image of eight Si(100) substrates placed onto the ceramic insert in the quartz boat. The metal housing and the white isolation material of the furnace can be seen on the right-hand side. The *blue frame* indicates the four substrates coated with TiO_2_ catalyst nanoparticles, and the *green frame*indicates four pristine substrates without catalyst. **b** Sketch of the substrate arrangement. Two substrates with (*blue*) and without catalyst (*green*) are placed next to each other. The ceramic insert assures the position of the substrates remains unchanged

Exact reproducibility of PVD growth experiments is a great challenge. One reason is the memory effect, i.e. that growth experiments are affected by deposits on the walls of the quartz tube from previous runs, and small variations in the growth parameters, such as temperature, resulting from slightly varied substrate arrangements. The catalyst-loaded and pristine substrates were overgrown simultaneously and in close proximity to one another to minimize these problems. Eight substrates were prepared for each growth run, out of which four were coated with TiO_2_ nanoparticles. The substrates are placed in the quartz boats before transfer into the furnace as shown in Fig. 1a. The Roman numerals from (I) to (IV) indicate pairs of substrates, whereby (I) corresponds to the hotter zone (close to the centre of the furnace) and (IV) to the colder downstream end. A ceramic insert supports the substrates as depicted in Fig. 1b so that their position is right in the vertical centre of the quartz tube in order to optimize the exposure to the vapour. Substrates next to each other are subject to the exactly same growth conditions, i.e. substrate temperature and flux.

**Fig. 2:**
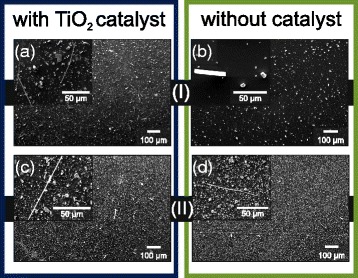
TiO_2_-catalyzed versus catalyst-free growth. SEM images of Bi_2_Te_3_ nanostructures from sample pairs from zones (I) and (II) with TiO_2_ catalyst nanoparticles (*blue frame*) and without catalyst (*green frame*). **a** Zone (I), TiO_2_: Platelets and belts grown with relatively high yield. Most platelets grow at an angle to the substrate normal. **b** Zone (I), catalyst-free: Only very few belts and almost no small platelets are grown. The most abundant structures are 10- *μ*m-diameter platelets. **c** Zone (II), TiO_2_: Dense growth with significantly increased density of belts. **d** Zone (II), catalyst-free: The density of platelets increases compared to **c**. Some thin nanowires are observed

After growth, all eight substrates are coated with a grey or silver-coloured layer judging from the optical inspection; samples with and without catalyst look the same. SEM images, however, reveal the impact of TiO_2_ on the growth. EDX analysis confirms the stoichiometry to be Bi_2_Te_3_ (see below for more details). Samples grown next to each other are compared in Fig. 2 using the numbering scheme introduced in Fig. 1a. The grey coating layer observed for samples in zone (I) is a result of the growth of platelets which can be seen for both samples grown at the same temperature in Fig. 2a, b, for TiO_2_ and without catalyst, respectively. The catalyst facilitates the growth of a higher density of platelets and smaller platelet dimensions. Platelets grown without catalyst are larger with dimensions typically between 10 and 20 *μ*m. Sub-micron belts are only rarely observed, but a few were spotted as shown in the inset in Fig. 2b. TiO_2_ increases the areal density of belts drastically, as can be already seen in the overview in Fig. 2a. An ensemble of several belts is shown in the inset in Fig. 2a. For lower substrate temperatures, downstream in zone (II), the stoichiometry is Bi_2_Te_3_ as well. The platelet density increases for both samples although the size of the platelets decreases (see Fig. 2c, d). A larger number of belts are found as well, effectively catalyzed by the TiO_2_ nanoparticles (Fig. 2c). The inset shows that the hexagonal platelets are not only lying flat on the surface but also extend from the surface under an angle. Few electron-transparent, i.e. very thin, belts are observed on the corresponding catalyst-free substrate (Fig. 2d). The platelets grow horizontally, as in case of the thin film growth (see Fig. 2d, inset). Their shapes reflect the inherent hexagonal crystal structure. This indicates that the growth at an angle to the substrate is the characteristic of the catalyzed process. The samples grown at a lower temperature in zone (III) (not shown) are very similar to the ones grown in zones (I) and (II) in that a mixed growth of platelets and belts is observed. In contrast, the two substrates grown in zone (IV) are covered with a layer of Te and no Bi_2_Te_3_ is found. To summarize, the temperature window for the growth of Bi_2_Te_3_ sub-micron belts, using a furnace temperature of 600 °C, is a substrate temperature of 450–510 °C, with close to optimum conditions for 480 °C.

**Fig. 3:**
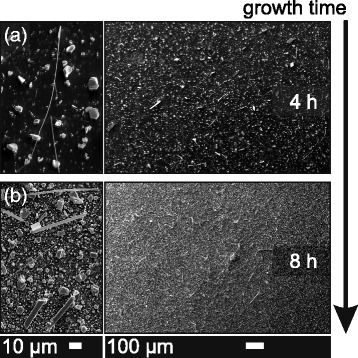
Time dependence of the growth. SEM images of two samples grown for **a** 4 h and **b** 8 h. The number of small platelets and the width of Bi_2_Te_3_ belts increase with growth time; however, the average length does not change significantly

Next, the dependence of belt dimensions on the growth time was studied, employing nanoparticle TiO_2_ catalyst. A growth time of 1 h was used for the catalyst comparison discussed above. The analysis of Fig. 2c yields an average length of belts of (53 ± 19) *μ*m. The distribution of dimensions across the ensemble is very heterogeneous, indicative of a random start time of the belt growth within the 1 h of growth. In Fig. 3a, we show an overview and a high-magnification image of a similar sample grown for 4 h. The average length is (107 ± 28) *μ*m, which is twice the average length for the 1-h sample. Larger and fewer platelets are found in Fig. 3a. For the 8-h sample, however, the number of small platelets increases (see Fig. 3b, high-magnification image) consistent with both previous samples grown at shorter growth times. The average length was estimated to (111 ± 50) *μ*m using the overview in Fig. 3b. This number is identical to the 4-h growth; however, the standard deviation has doubled. The reason for the increase in standard deviation instead of average length is twofold. Some belts have grown for the entire duration to become extremely long, whilst short ones have just started to grow when the growth period is finished. Furthermore, the average width (neglecting rotation with respect to the viewing direction) has increased from (2.0 ± 0.9) to (3.4 ± 1.2) *μ*m indicating horizontal growth where the material is deposited onto the sidewalls. So far, it is not clear what determines whether the vertical (on top of the layers) or horizontal growth dominates.

**Fig. 4:**
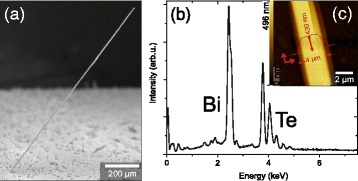
Millimetre-long sub-micron belt. **a** SEM image of a free-standing Bi_2_Te_3_ belt. **b** EDX analysis shows the composition to be stoichiometric Bi_2_Te_3_. **c** AFM image of the long Bi_2_Te_3_ belt, with the height profile in *red*, showing a thickness of 430 nm and a width of 4 *μ*m, i.e. an aspect ratio of thickness to width of ∼1:10

Typically, over a substrate area of 1 × 1 cm^2^, one to two very long structures can be found, as shown in Fig. 4a. The free-standing structure has a length of at least 0.8 mm, a width of 4 *μ*m, and a thickness of 430 nm. It stands without bending at a height-to-width ratio of only ∼1:10 and a height-to-length ratio of ∼1:2000. Assuming a constant growth scenario over the 8 h of growth, the growth rate was larger than 28 nm/min. The composition was determined to be stoichiometric Bi_2_Te_3_ using EDX, as shown in Fig. 4b. Subsequent to the growth, the belt was transferred by hand using a micro-needle and deposited onto a Si wafer to study its height profile using AFM (see Fig. 4c). The observation of step edges is an indicator of a growth direction parallel to the QLs.

**Fig. 5:**
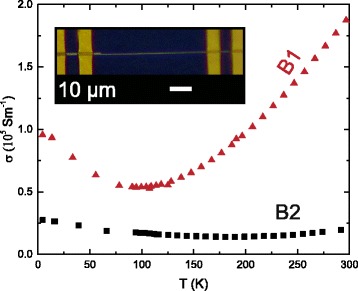
Electrical characterization of a long sub-micron belt. The measured temperature-dependent electrical conductivity *σ*(*T*) of two belts from the same batch as function of the bath temperature *T*. The *inset* shows a microscope image of the contacted B2 allowing for measurements in four-point configuration. *Yellow*: Au, *purple*: SiO_2_, *green*: Bi_2_Te_3_ belt

Similar belts, labelled B1 and B2, were picked from the same substrate for four-point-probe measurements. A typical device is shown in the inset of Fig. 5. The distance *l* between the voltage probes is *l*_B1_=31.4 *μ*m and *l*_B2_=52.5 *μ*m for B1 and B2, respectively.

The resistance *R* was studied as a function of temperature *T*. The electrical conductivity *σ*(*T*) of each belt was calculated using

1 $$ \sigma({T}) = \frac{1}{R({T})} \frac{l_{\mathrm{B}}}{A_{\mathrm{B}}},   $$

with *A* the belt’s rectangular cross section given by *A*_B_=*h*_B_×*w*_B_ (see Fig. 4(c) for qualitative comparison). The room temperature values for *σ* and the dimensions of B1 and B2 are given in Table 1.

**Table 1:** Summary of the electrical conductivity *σ* at room temperature, the belt width *w*_B_, height *h*_B_, and length of the central part *l*_B_, and the surface-to-volume-ratio *S*/*V*

*σ*	*w*_B_	*h*_B_	*l*_B_	*S*/*V*	Sample
(10^5^ S m^−1^)	(nm)	(nm)	(*μ*m)	(10^7^ m^−1^)	
0.20	246	261	31.4	1.58	B1
1.88	514	131	52.5	1.92	B2

B1 has a conductivity of ∼0.5×10^5^ S m^−1^, comparable to the low value reported for *n*-type bulk Bi_2_Te_3_ [21]. The electrical conductivity of the two belts differs by nearly one order of magnitude. Stoichiometry variations beyond + 2 % Te in Bi_2_Te_3_, as determined by EDX on several belts, can be excluded as the cause of this difference. Further, deviations in electrical conductivity can originate from defects or Te-depletion near the surface that leads to a surface layer of high electrical conductivity [22]. In our case, the surface-to-volume-ratio of B2 is about 20 % higher than that of B1 so that surface effects may be, at least in parts, the origin of the higher electrical conductivity of B2 [4].

The temperature-dependent electrical conductivity is shown in Fig. 5. Both belts show a minimum in the conductivity at an intermediate temperature of 185 K for B1 and 95 K for B2, respectively. A characteristic minimum in the conductivity has also been observed in Bi_2_Te_3_ bulk samples and nanostructures grown by different methods [23–27]. The feature appears at a temperature when the contribution of the surface conduction to the total conductivity becomes significant compared to the bulk contribution at a carrier density below 1×10^17^cm^−1^. Further studies will employ magnetoresistance measurements at low temperatures to distinguish between both contributions.

## Conclusions

In summary, we have studied the growth of Bi_2_Te_3_ sub-micron belts using TiO_2_ nanoparticles as catalyst. The growth on substrates coated with the catalyst solution was compared to pristine Si substrates, overgrown under exactly the same conditions. The catalyst-coated substrates have a much higher belt yield; however, self-catalysed growth is also present. Very long belts can be grown from TiO_2_, with their length only limited by the growth time. For an 8-h growth, belts of up to ∼1 mm in length were produced. Their exceptional length makes these belts suitable candidates for electronic transport studies. The conductivity is as low as for pure bulk Bi_2_Te_3_ which is an advantage for the observation of the topological surface state and a sign of excellent crystal quality. Future work may explore the correlations between thermoelectric properties and the topological surface state further for an application of Bi_2_Te_3_ nanowires as versatile building blocks for thermoelectric, sensor, and spintronic devices.

## Abbreviations

AFM: atomic force microscopy
EDX: energy-dispersive X-ray spectroscopy
FIB: focused ion beam
PVD: physical vapour deposition
SEM: scanning electron microscopy
TI: topological insulator
TSS: topological surface state
VLS: vapour-liquid-solid
